# Abstract of Proceedings of Buffalo Medical Association, Jan. 2d, 1877

**Published:** 1877-03

**Authors:** 


ART. II.—*Abstract of the Proceedings of the Buffalo Medical Association, January 2d,* 1877.

Members present—the President, Dr. Wyckoff, in the chair, Drs. Rochester, Miner, White, Samo, S. S. Greene, Bartlett, Brecht, Howe, Brush, W. W. Miner, Fowler and Dorr.

The essayist for the evening, Dr. Barnes, was absent, and no paper was presented.

DR. ROCHESTER reported the case of a young man aged 25, weight last fall 98 pounds, who recently died suddenly of heart disease. He had been a patient of his for seven or eight years, having had during that time several attacks of rheumatism. He had also had pleuritis and subsequently pneumonia.

A *post mortem* examination was made, which revealed the pericardium wholly adherent to the heart and considerably thickened. The adhesion was so strong that in some places it could only be separated with great difficulty. The heart was removed and found to weigh twenty-nine ounces. It was estimated that at the time of death the patient could not have weighed more than from one hundred and seven to one hundred and ten pounds.

The mitral valve was the seat of ossific deposit, and there was also a slight lesion of the aortic valve. He never knew that the patient had pericarditis, but from the fact that pleuritis had been present and that the pleura was adherent to the pericardium he presumed the inflammation had extended from one membrane to the other.

DR. WHITE said that he was present at the *post mortem* referred to, and to him the condition was an interesting revelation. He would like Dr. Rochester to explain the cause of death.

DR. ROCHESTER replied that in hypertrophy of the heart death was often sudden and inexplicable. He had in his cabinet a heart which weighed, when removed, sixty ounces, and at the *post mortem* which included an examination of all the organs, no satisfactory cause of death could be found. (Here followed some general conversation in regard to heart disease which the Secretary was unable to take down.)

DR. S. S. GREENE reported a case of retention of urine caused by a calculus being lodged in the urethra, which he could only extract by resorting to the urethrotomy. He was doubtful as to the origin of the stone, whether in the bladder or in the urethra, if in the former its size would show that considerable distension had taken place in the neck of the bladder to allow its passage.

Dr. Miner said he had a case now under observation in which, in the course of a year, an ounce of small calculi would be voided from the bladder with the urine, sometimes demanding assistance in their passage, but generally passing without difficulty.

Dr. Howe presented the history in brief of a case of retinal glioma. He exhibited the eye after removal, and also two casts of the patient's face, one at the time of operation, the other subsequent to death. The patient was a little girl who presented the disease in an advanced stage, the operation was made with an idea of relieving pain and prolonging life. One month after the operation the patient died.

Dr. Miner said that the matter of recurrence in these cases was one which was to be taken into consideration. The case reported by Dr. Howe was one in which it was exceedingly proper to operate, but generally these cases proved fatal by a recurrence of the disease. This was, unfortunately, the case also in malignant diseases situated in other localities, the breast, the vulva, and the uterus, in many cases the disease returned in the original locality or in some other, and proceeded to a fatal termination.

Dr. Howe said that in regard to the eye he thought there were two reasons why the operation was advisable: 1st. Relief from pain. In the case reported, and in others of a similar nature, the origin of the disease was in the retina, and as it progressed the unyielding nature of the sclerotic coat, which would not give way as readily as some other tissues, gave rise to intense pain. 2d. In some cases the patient recovered. He presented nearly a year ago, a patient to the society with retinal glioma, which had to be subsequently removed, and up to date there had been no signs of return.

Dr. White said that so important a topic could not be discussed intelligently on short notice. He thought Dr. Miner too skeptical, that the breast could be removed for cancer, or the neck of the uterus amputated, and prolong the life of the patient for many years. Without prolonging the discussion, he thought it a very proper subject for special discussion at some subsequent meeting. He desired to report a case which afforded the topic for some considerations in reference to the treatment of ovarian cysts of small size, when situated in the cul de sac of Douglas. Not long since he was called to treat a patient in an eastern county of this state. Without entering into her history, he would simply state that she complained of pressure on the rectum, a sense of fullness in the pelvis. The uterus was carried up and forward, the bladder somewhat pressed upon. A cyst was plainly made out, and its contents evacuated through the vagina. The fluid removed was semi-gelatinous and coagulated at once on the application of heat. The tumor was found to be multilocular. The cysts were evacuated and injected with tincture of iodine. The result he was not prepared to announce. He had been informed that a slight peritonitis had followed, but nothing alarming. He had had, in his own practice and in consultation, perhaps half a dozed similar cases, and had treated them in various ways by removal through the vagina and by injection. He had removed but one by sections, through the vagina, the pedicle was separated by the ecraseur. He brought up the subject to get the opinion of the members as to the best method of dealing with similar cases.

Dr. Miner said that in the *Boston Med. Journal* of Nov. 2d, was the report of a case by Dr. Clifton Wing, in which Dr. Warren had removed the tumor through the vagina by enucleation.

Dr. Rochester said that two cases had come under his observation. The first was that of a laboring woman, who complained of a pain on defacation, a sense of fullness in the pelvis, etc. Under the idea that it might be an hæmatocele, he introduced a trocar and drew off the contents, injecting two or three ounces of glycerine with carbolic acid, four grains to the ounce. A complete curve was the result. In the second case the tumor was simply tapped, but the patient was lost sight of.

Dr. Miner said that while talking on this subject, he wished to report two cases of ovarian tumor. One, a lady in Canada, has at intervals a discharge of the contents of an ovarian cyst through the rectum. The tumor subsequently filling up and again discharging. The other was a lady that came down to him from Cattaraugus County, with a fistula in the side, through which the contents of an ovarian cyst had spontaneously discharged. He introduced an elastic catheter through the opening and washed out the cavity with water containing a trace of carbolic acid. This was done daily for some time until a cure resulted. The discharge ceased, the opening closed and the tumor has not since appeared. Its size was such that at the first visit she made him, on enlarging the opening and introducing a tube, a large wooden pailful of fluid was removed.

DR. BRUSH reported a case of Syphilitic Disease of the Brain under his care at the Hospital of the Sisters of Charity, which was convalescent under large doses of Iodide of Potassium.

DR. SAMO reported a case of a child, probably syphilitic, in which epileptiform convulsions yielded to Iodide of Potassium.

DRS. S. S. GREEN and BARTLETT also reported cases illustrative of the value of Potassium Iodide in similar cases, and sometimes in establishing a diagnosis.

On motion, the Association adjourned.

